# *Giardia* co-infection promotes the secretion of antimicrobial peptides beta-defensin 2 and trefoil factor 3 and attenuates attaching and effacing bacteria-induced intestinal disease

**DOI:** 10.1371/journal.pone.0178647

**Published:** 2017-06-16

**Authors:** Anna Manko, Jean-Paul Motta, James A. Cotton, Troy Feener, Ayodele Oyeyemi, Bruce A. Vallance, John L. Wallace, Andre G. Buret

**Affiliations:** 1 Department of Biological Sciences, University of Calgary, Calgary, Alberta, Canada; 2 Inflammation Research Network, University of Calgary, Calgary, Alberta, Canada; 3 Host-Parasite Interactions, University of Calgary, Calgary, Alberta, Canada; 4 Department of Pediatrics, Division of Gastroenterology, Child and Family Research Institute, Vancouver, British Columbia, Canada; 5 Department of Physiology & Pharmacology, University of Calgary, Alberta, Canada; Aga Khan University Hospital Nairobi, KENYA

## Abstract

Our understanding of polymicrobial gastrointestinal infections and their effects on host biology remains incompletely understood. *Giardia duodenalis* is an ubiquitous intestinal protozoan parasite infecting animals and humans. Concomitant infections with *Giardia* and other gastrointestinal pathogens commonly occur. In countries with poor sanitation, *Giardia* infection has been associated with decreased incidence of diarrheal disease and fever, and reduced serum inflammatory markers release, via mechanisms that remain obscure. This study analyzed *Giardia spp*. co-infections with attaching and effacing (A/E) pathogens, and assessed whether and how the presence of *Giardia* modulates host responses to A/E enteropathogens, and alters intestinal disease outcome. In mice infected with the A/E pathogen *Citrobacter rodentium*, co-infection with *Giardia muris* significantly attenuated weight loss, macro- and microscopic signs of colitis, bacterial colonization and translocation, while concurrently enhancing the production and secretion of antimicrobial peptides (AMPs) mouse β-defensin 3 and trefoil factor 3 (TFF3). Co-infection of human intestinal epithelial cells (Caco-2) monolayers with *G*. *duodenalis* trophozoites and enteropathogenic *Escherichia coli* (EPEC) enhanced the production of the AMPs human β-defensin 2 (HBD-2) and TFF3; this effect was inhibited with treatment of *G*. *duodenalis* with cysteine protease inhibitors. Collectively, these results suggest that *Giardia* infections are capable of reducing enteropathogen-induced colitis while increasing production of host AMPs. Additional studies also demonstrated that *Giardia* was able to directly inhibit the growth of pathogenic bacteria. These results reveal novel mechanisms whereby *Giardia* may protect against gastrointestinal disease induced by a co-infecting A/E enteropathogen. Our findings shed new light on how microbial-microbial interactions in the gut may protect a host during concomitant infections.

## Introduction

*Giardia duodenalis* (syn. *G*. *lamblia*, *G*. *intestinalis)* is a ubiquitous intestinal protozoan parasite that infects a wide array of hosts, and is responsible for diarrheal disease as well as numerous post-infectious extraintestinal pathologies [1–5]. It is one of the most common fecal-oral parasitic infection of the human small intestine worldwide [1, 4, 5]. Due to the high burden of *G*. *duodenalis*-related illness on health and economics in the developing world, this protozoan parasite has been included in the World Health Organization’s (WHO) Neglected Diseases Initiative since 2006 [6, 7]. It is estimated that more than one billion people are at risk of infection from *G*. *duodenalis* each year [8]. *Giardia* infection can be an asymptomatic, or cause acute self-limiting diarrhea or chronic diarrhea, with or without dehydration, and with or without intestinal malabsorption [9–11]. Giardiasis is responsible for failure to thrive and cognitive malfunction in children from areas of the world where the infection is endemic [1, 12]. In spite of high parasite loads that can exceed 10^6^ trophozoites per centimeter of gut during the acute stage of the infection, the intestinal mucosa of *Giardia*-infected hosts is devoid of overt signs of inflammation [4, 13–15].

*Giardia* infections are acquired via ingestion of infectious cysts in contaminated food or water sources, or directly via the fecal-oral route. These routes of infection are shared among a broad variety of gastrointestinal (GI) pathogens, and as a result, *Giardia* co-infections are common, especially in regions with poor water and food sanitation [16]. As discussed in a recent editorial, more research needs to characterize how concurrent infections may alter disease outcome, either directly or indirectly [17]. Using complex polymicrobial infection model systems that mimic true disease conditions will help uncover new, more realistic, therapeutic targets. *Giardia* infections have been reported concomitantly with bacterial, viral, and/or other parasitic enteropathogens [18–25]. Even though most of these pathogens are known to cause diarrheal disease, some reports suggest that *Giardia* may attenuate diarrheal illness severity [16, 26]. The mechanisms are unknown. Similarly, clinical research in those parts of the world has shown that children infected with *G*. *duodenalis* have a reduced likelihood of developing diarrheal disease and fever, and have reduced serum inflammatory scores compared to *Giardia*-negative children [27, 28]. In Bangladesh, *Giardia* infections have been associated with the development of diarrhea [29], yet, other studies in children from Bangladesh have suggested that the parasite neither increased nor decreased the odds of acute diarrhea [30]. Other reports indicate that Tanzanian children infected with *Giardia* were less likely to develop diarrheal disease when compared to children not infected with *Giardia* [27]. More research is required to elucidate how *Giardia* infections may result in diarrheal disease or remain asymptomatic.

Other reports have suggested that *Giardia* may be deleterious during co-infections with other GI pathogens [31, 32]. While many studies have investigated host immunity in giardiasis, it remains to be shown whether and how *Giardia* may have immunomodulatory effects that could modulate host susceptibility to GI co-infections [33].

The production of antimicrobial peptides (AMPs) by intestinal epithelial cells (IECs) plays a critical role in intestinal mucosal homeostasis and antimicrobial immunity against enteropathogens [34]. *Salmonella spp*., *Shigella spp*., *Citrobacter rodentium*, enteropathogenic *Escherichia coli* (EPEC) and enterohemorrhagic *E*. *coli* (EHEC) are able to induce the production of AMPs in humans or mice [35–37]. For example, in humans, human β-defensin-2 (HBD-2) is induced in IECs *in vitro* following exposure to pathogenic *Salmonella spp*. infection [38, 39] and in gastric epithelial cells, during human *Helicobacter pylori* infection [38]. HBD-2 has microbicidal activity against Gram positive and negative bacteria and yeast [40]. The murine homolog of HBD-2, mouse *β*-defensin 3 (mBD3), is broadly expressed within murine GI tissues, and has broad-spectrum antimicrobial activity [41, 42]. Trefoil factor 3 (TFF3) is a small cysteine-rich secretory peptide that is expressed by goblet cells throughout the gastrointestinal tract [43]. TFF3 promotes epithelial restitution following mucosal injury [44] and enhances the protective barrier properties of the mucus layer, which is important for protection against enteropathogens [45]. While not having a direct antibacterial effect, it protects the host against infection by binding to bacteria, and by synergizing with Muc2 [46–49]. The role of AMPs during *Giardia* infections remains incompletely understood. Previous experiments performed *in vitro* have demonstrated that *G*. *duodenalis* trophozoites are susceptible to intestinal epithelial AMPs [50], and intestinal epithelial cells exposed to *Giardia* parasites *in vitro* enhance production of matrix metalloprotease 7, mediators that are required for the activation of α-defensins [51]. Whether and how *Giardia* may affect AMP production in IECs to modulate disease during co-infections has yet to be assessed.

The *Giardia* genome contains 27 cathepsin B- and L-like cysteine proteases [52], and several of these proteases are released upon exposure to epithelial cells *in vitro* [53]. Our understanding of *Giardia* cathepsin cysteine proteases and their role during host-parasite interactions is ever expanding. Previous research has demonstrated that *Giardia* cathepsin cysteine proteases are implicated, at least in part, in trophozoite encystation and excystation [54], degradation of pro-inflammatory chemokines [26], and some of the pathophysiological changes observed in infected enterocytes [55]. The role of *Giardia* proteases in the modulation of AMPs and enteric disease modulation during co-infections is unknown.

Recent findings have established that *Giardia* infection may have detrimental effects at intestinal sites beyond its area of trophozoite colonization, including post-infectious hypersensitivity in the rectum [12] and goblet cells depletion in the colon [56]. The mechanisms remain unclear, but these observations provide a rationale for investigating the effects of *Giardia* during co-infections with enteropathogens in colonic cells and tissues.

Together, the data describe a new causal relationship between the presence of *Giardia* and the attenuation of intestinal disease caused by a co-infecting A/E entropathogen.

## Results

### *G*. *muris* infection attenuates *C*. *rodentium*-induced colitis

**Fig 1:**
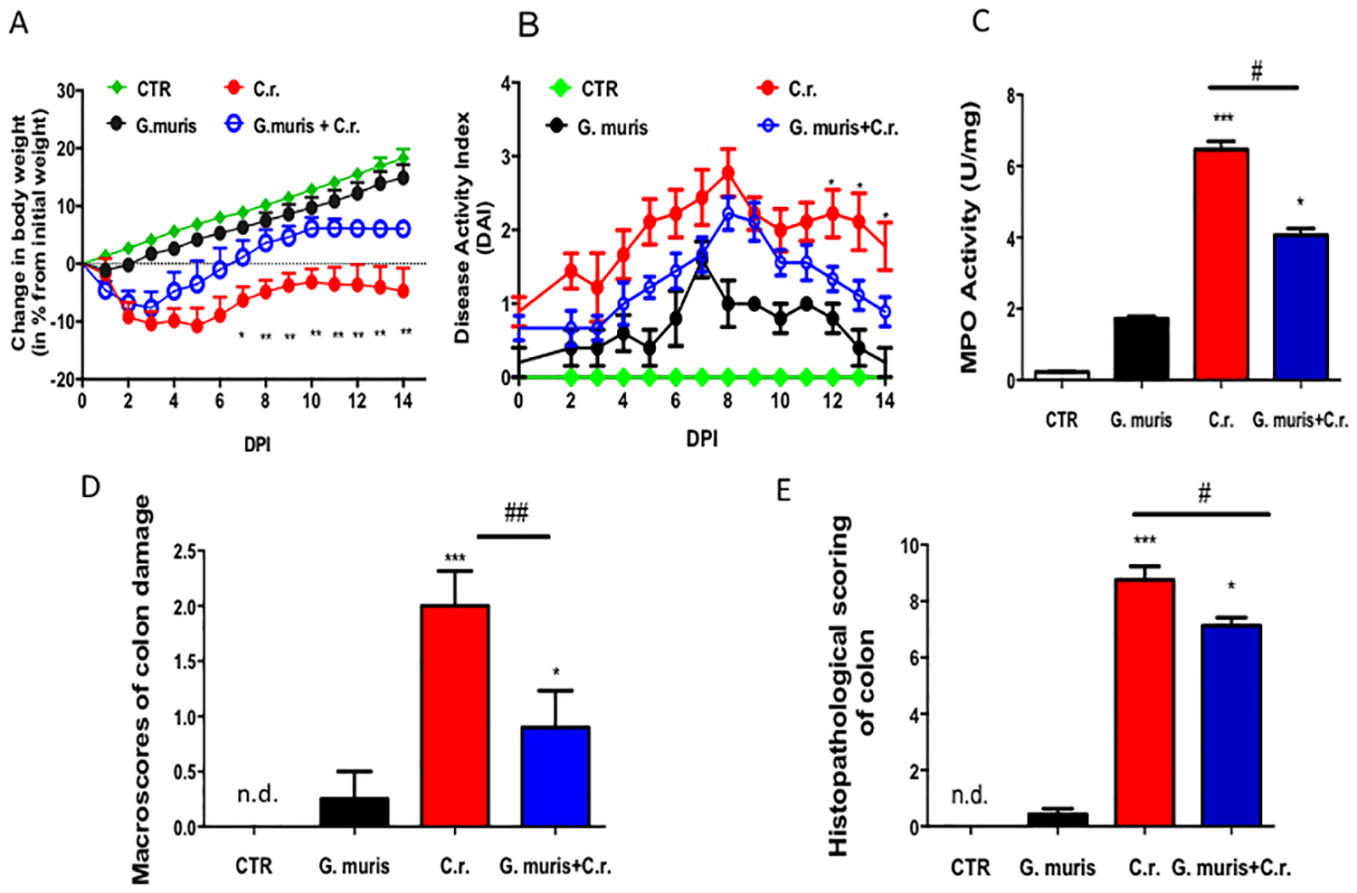
*G*. *muris* attenuates concomitant *C*. *rodentium*-induced colitis. Male C57/BL6 mice (7–8 week old) were infected with either *G*. *muris*, or *C*. *rodentium (C*.*r*.*)*, or both in co-infection (*G*. *muris + C*.*r*.*)*. The figure illustrates (A) Changes in body weights and (B) disease activity index (DAI) assessed over 14 days. (C) On day 14 post-infection, colonic myeloperoxydase activity (MPO), (D) macroscopic damage of the colon and (E) histopathological damage scores on H&E stained tissue were assessed. All data are representative of n = 5–10/group and represented as mean±SEM. * p<0.05, ** p<0.01, *** p<0.001 –compared to control group (CTR); # p<0.05, ## p<0.01 versus the corresponding group, indicated by line; n.d. means not detected, that is equivalent to 0.

Our initial experiments sought to determine whether *Giardia* infections were protective during co-infection with the A/E pathogen *C*. *rodentium*. Therefore, 7 to 8 week old mice were co-infected with *G*. *muris* and *C*. *rodentium* for 14 days. The murine enteropathogen *C*. *rodentium* is commonly used a model for EPEC and EHEC infections [57, 58]. Similarly, *G*. *muris* is often used to model human *G*. *duodenalis* infections [59, 60]. Animals infected with *C*. *rodentium* lost weight from the first day of infection and did not return to baseline weight (Fig 1A), while animals co-infected with *G*. *muris* and *C*. *rodentium* lost weight at the beginning of infection but then dramatically recovered weight gain by day 7 compared to mice infected with *C*. *rodentium* alone (Fig 1A). Control animals and animals infected with *G*. *muris* gained weight similarly throughout the experimental time (Fig 1A). In co-infected animals, disease activity index (DAI) scores (fecal blood, fecal consistency, and histological evidence of erythema edema, colonic thickness) were higher than in control animals (Fig 1B), but significantly lower than in animals infected with *C*. *rodentium* alone (Fig 1B).

*C*. *rodentium* increased MPO activity in colonic tissue levels versus control values (Fig 1C). Co-infection with *G*. *muris* significantly attenuated this effect (Fig 1C). *G*. *muris* alone did not change colonic MPO activity (Fig 1C). Infection with *C*. *rodentium* alone also significantly increased colonic macro- and microscopic damage scores compared with uninfected control animals (Fig 1D and 1E, respectively). Co-infection with *G*. *muris* significantly attenuated this effect (Fig 1D and 1E). Damage scores from tissues of mice infected with *G*. *muris* alone were similar to controls (Fig 1D and 1E). Histopathology revealed that *C*. *rodentium* caused significant microscopic damage in the colon at 14 DPI (S1 Fig). Tissues from animals co-infected with *G*. *muris* showed only mild mucosal inflammatory infiltrates and edema (S1 Fig).

### *G*. *muris* infection reduces fecal *C*. *rodentium* numbers

**Fig 2:**
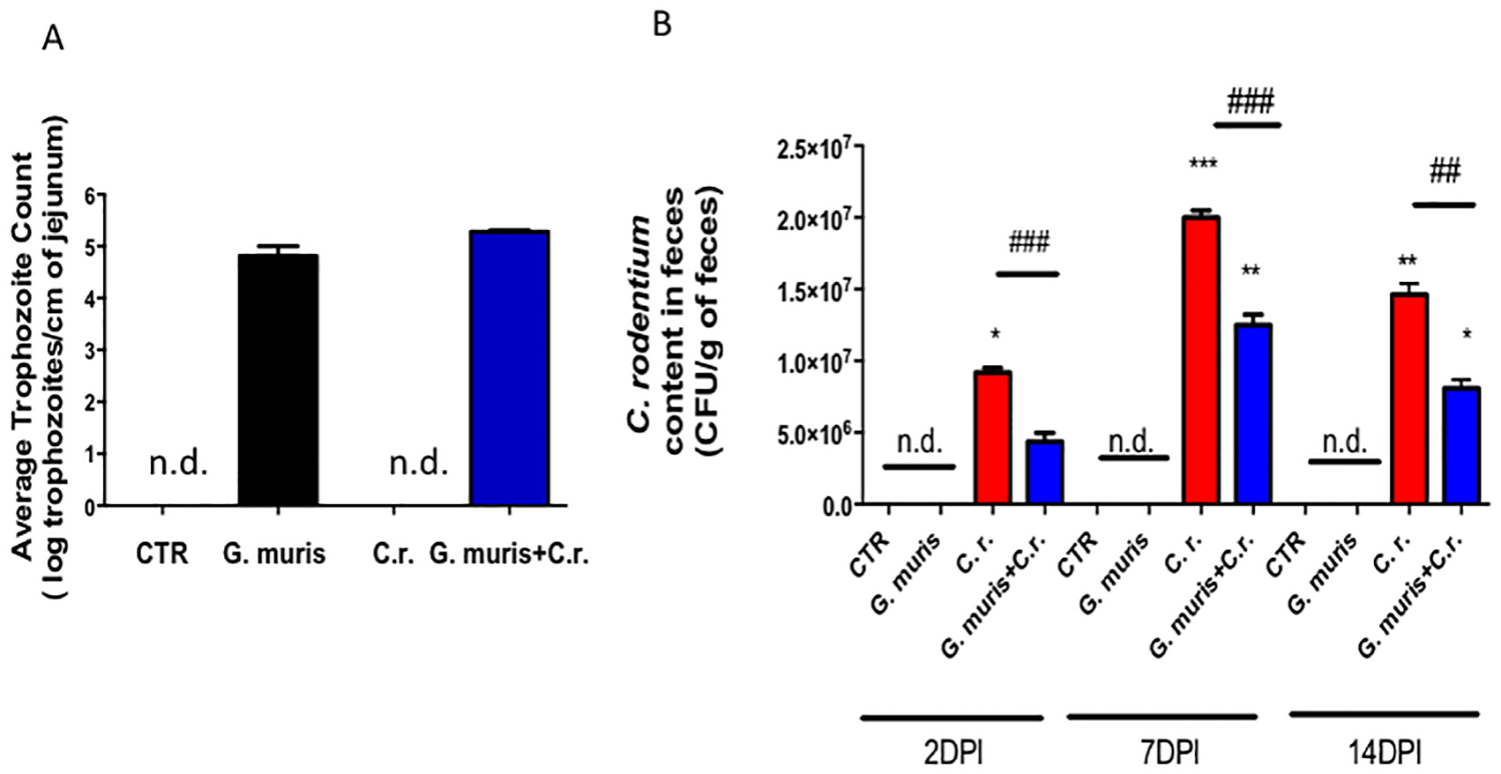
Duodenal *G*. *muris* trophozoite counts, and fecal *C*. *rodentium* burden. Male C57BL/6 mice aged 7 to 8 weeks were infected with *G*. *muris* and *C*. *rodentium (C*.*r*.*)* separately and in co-infection (*G*. *muris* + *C*.*r*.) (A) On day 14 post- infection, numbers of *G*. *muris* trophozoites were counted in the jejunum. (B) *C*. *rodentium* fecal burden was determined by plating colonic stool contents on Lysogeny broth (LB) agar plates. All data are representative of n = 5–10/group and represented as mean±SEM. * p<0.05, ** p<0.01, *** p<0.001– compared to control group (CTR); ### p<0.001 versus the corresponding group, indicated by line; n.d. means not detected, that is equivalent to 0.

We next sought to determine whether the differences in the severity of colitis were associated with alterations of the gut microbial burden. On day 14 post-infection, trophozoites were enumerated in the jejunum of both groups infected with *Giardia* and values were not significantly different from each other (Fig 2A). In contrast, *C*. *rodentium* fecal CFUs were significantly lower in animals co-infected with *G*. *muris* than in those infected with *C*. *rodentium* alone (on days 2 and 7 post-infection) (Fig 2B).

### *Giardia* prevents attachment and translocation of *C*. *rodentium* into host tissues

**Fig 3:**
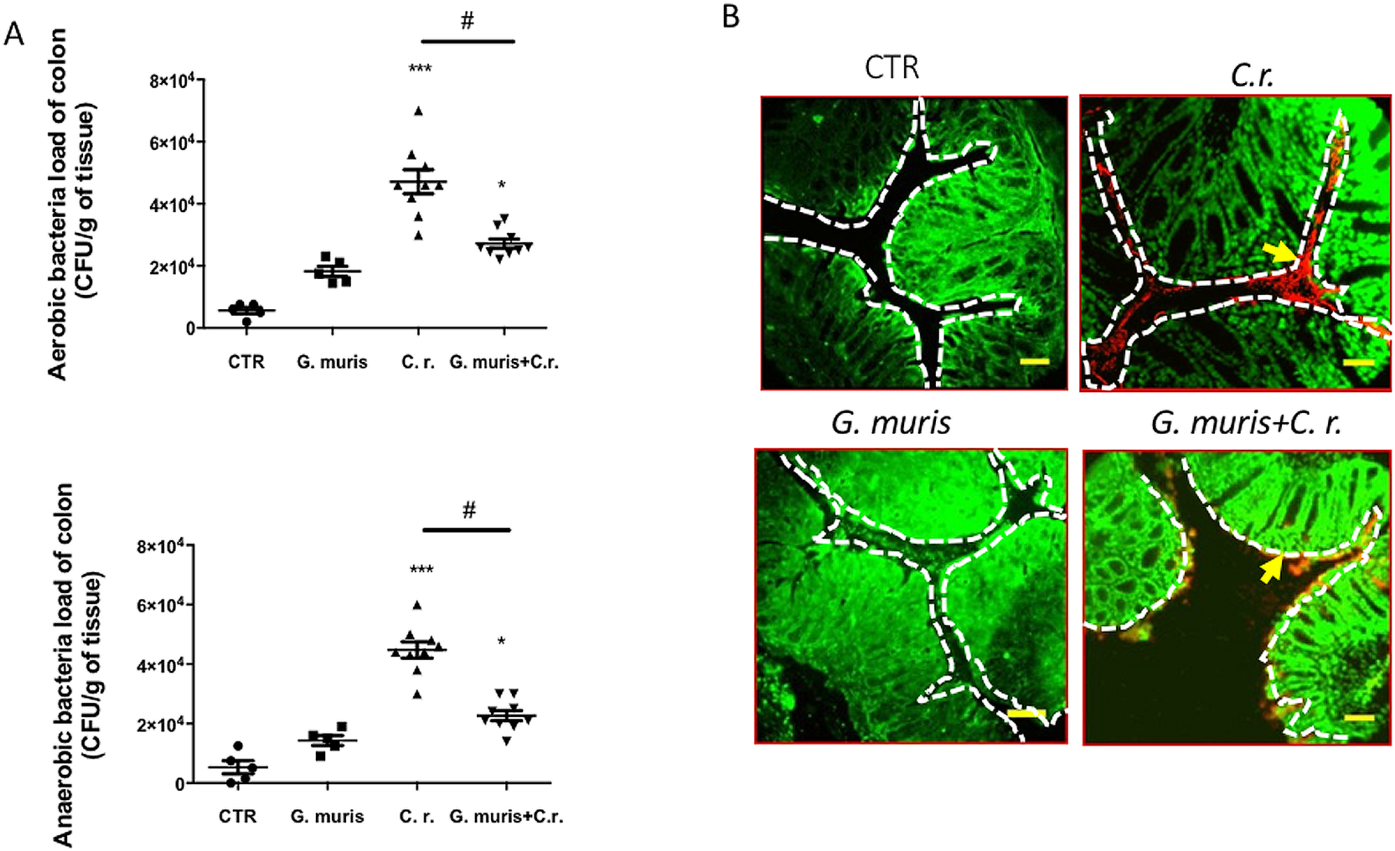
*G*. *muris* reduces *C*. *rodentium* colonization in the colon of co-infected mice, and decreases bacterial attachment to colonic mucosa. Male C57BL/6 mice aged 7 to 8 weeks were infected with *G*. *muris* and *C*. *rodentium (C*.*r*.*)* separately and in co-infection (*G*. *muris* + *C*.*r*.) (A) On 14^th^ day post-infection colon samples were collected and homogenized for aerobic and anaerobic colonic bacterial loads. (B) *C*. *rodentium* in the colon was stained using anti-lipopolysaccharide (LPS). The surface of the colonic mucosa is indicated by a white dashed line, and arrows indicate bacterial attachment to the colonic mucosa (Original magnification = 400x. Scale bar = 25 μm. Red—bacteria, Green—DAPI). All data are representative of n = 5–10/group and represented as mean±SEM. * p<0.05, ** p<0.01, *** p<0.001– compared to control group (CTR); # p<0.05 versus the corresponding group, indicated by line.

Follow-up analyses set out to determine whether *G*. *muris* co-infection prevented *C*. *rodentium* from disseminating into host tissues. Both aerobic and anaerobic bacterial loads of colonic homogenates were significantly reduced in the co-infected group compared to *C*. *rodentium*-infected mice (Fig 3A). In order to selectively visualize *C*. *rodentium* in the colon *in situ*, we used a green-fluorescent protein (GFP)-expressing *C*. *rodentium*, and immunostaining for *C*. *rodentium* lipopolysaccharide (LPS) [61]. In animals infected with *C*. *rodentium* alone, large numbers of bacteria were detected in the lumen, attached to the mucosa, and within colonic tissues. In mice co-infected with *G*. *muris*, *C*. *rodentium* was occasionally attached to the mucosa but was mostly localized in the lumen and not seen within tissues (Fig 3B and S2 Fig). Staining of *C*. *rodentium* LPS demonstrates bacterial attachment to the epithelial surface (Fig 3B), which was confirmed by staining of GFP-labelled *C*. *rodentium* (S2 Fig). This technique also revealed translocation of bacteria within the colonic tissue.

**Fig 4:**
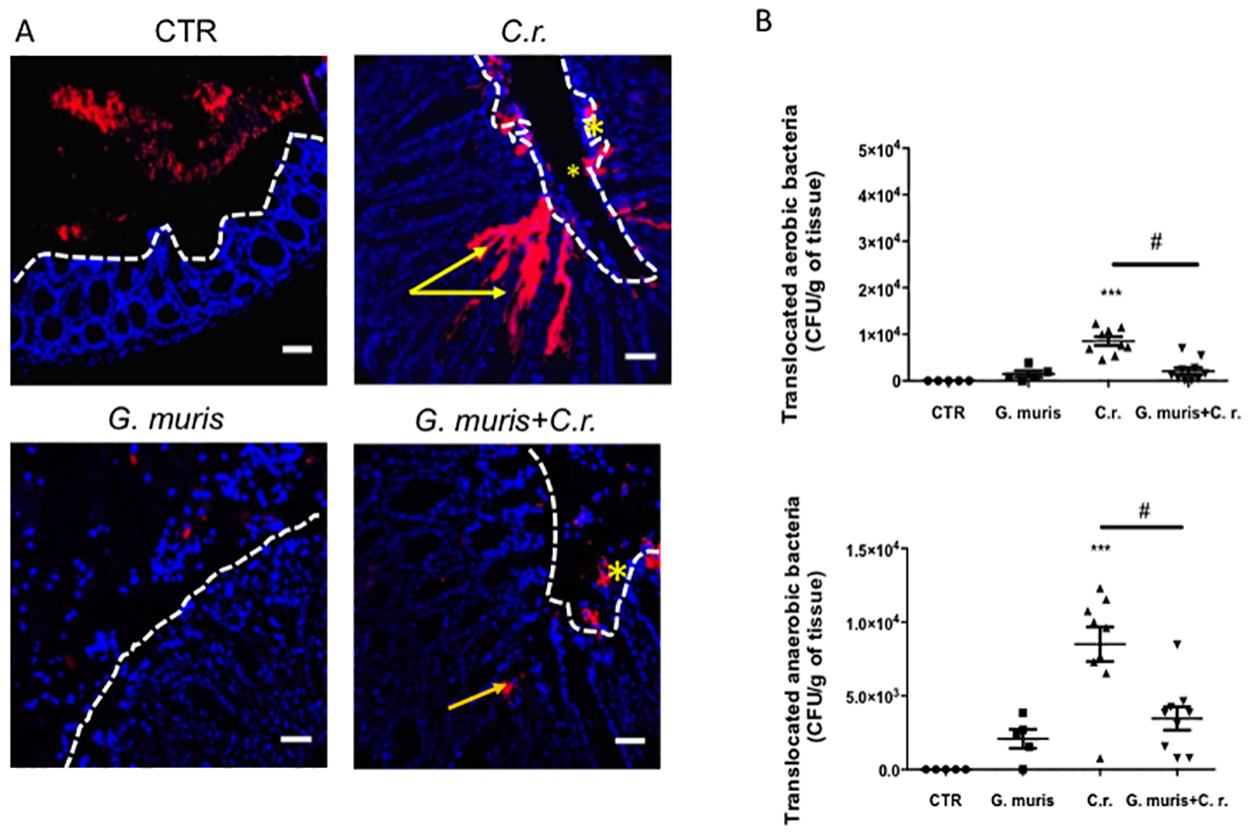
*G*. *muris* reduces *C*. *rodentium* attachment to colonic tissues, and translocation to the spleen. Male C57/Bl6 mice (7–8 week old) were infected with *G*. *muris* and *C*. *rodentium (C*.*r*.*)* separately and in co-infection. On day 14 post-infection colon samples and spleen were collected and analyzed. (A) Fluorescent *in situ* hybridization (FISH) staining was performed on the colonic samples. (Original magnification = 400x. Scale bar = 25 μm. Red—bacteria, Blue—DAPI). Stars identify bacteria attached to mucosa, and arrows indicate bacteria that have translocated into the lamina propria. (B) Translocation of aerobic and anaerobic bacteria to the spleen. All data are representative of n = 5–10/group and represented as mean±SEM. * p<0.05, ** p<0.01, *** p<0.001– compared to control group (CTR); # p<0.05 versus the corresponding group, indicated by line.

We next used fluorescent *in situ* hybridization (FISH) to visualize the translocation of commensal microbiota bacteria into tissues underlying the colonic epithelium. In uninfected mice, commensal microbes were kept at distance from the epithelium, and bacterial translocation into the lamina propria was not observed (Fig 4A). In animals infected with *C*. *rodentium* alone, commensal bacteria were observed deep in the bottom of intestinal crypts and within the mucus layer (Fig 4A). In mice co-infected with *G*. *muris*, the deeper invasion of microbiota was not observed (Fig 4A). Another set of experiments sought to determine whether *G*. *muris* was able to reduce *C*. *rodentium*-induced translocation of bacteria to distant organ sites. Infection with *C*. *rodentium* alone significantly elevated the numbers of aerobic and anaerobic bacteria translocated to both the spleen and liver (Fig 4B and S3 Fig). In mice co-infected with *G*. *muris*, aerobic and anaerobic bacterial counts in the spleen and liver were significantly reduced (Fig 4B and S3 Fig). Infection with *G*. *muris* alone induced a low degree of bacterial translocation (Fig 4B and S3 Fig).

### *Giardia* stimulates transcription and expression of β-defensin 3 (MBD-3) and Trefoil factor 3 (TFF3)

**Fig 5:**
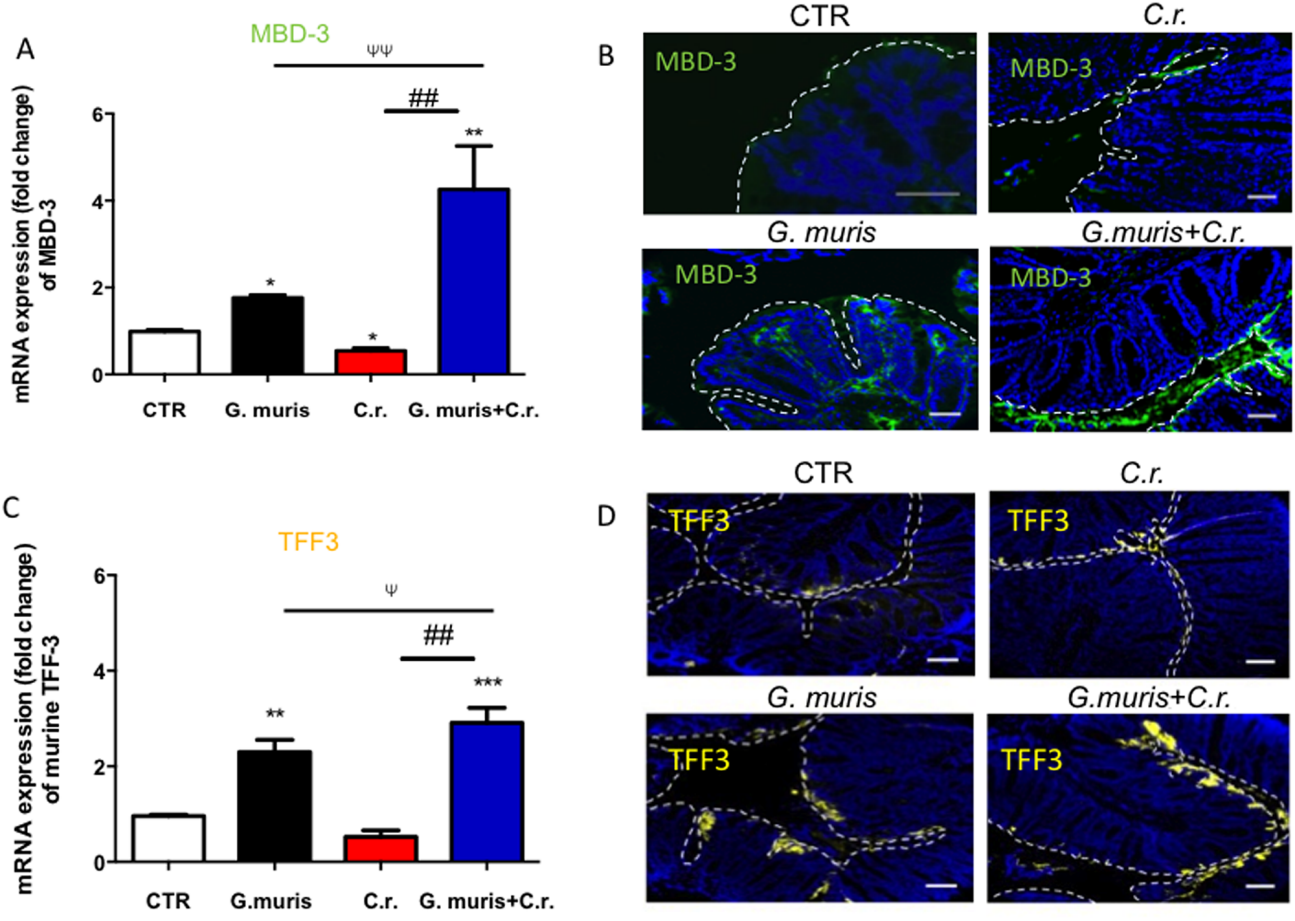
Animals co-infected with *G*. *muris* and *C*. *rodentium* have elevated colonic expression and secretion of MBD-3 and TFF-3. Male C57/BL6 mice (7–8 week old) were infected with *G*. *muris* and *C*. *rodentium (C*.*r*.*)* separately and in co-infection. The figure illustrates results from analyses of the colon performed 14 days post-infection. (A and C) Colonic mRNA levels of mbd-3 and tff3 were assessed. (B and D) Immunofluorescence of MBD-3 (green) and TFF3 (yellow) was also performed. Original magnification = 400x. Scale bar = 25 μm. Colonic mucosa is indicated by a white dashed line. All data are representative of n = 5–10/group and represented as mean±SEM. * p<0.05, ** p<0.01, *** p<0.001– compared to control group (CTR); ## p<0.01 versus the corresponding group, indicated by line; Ψ p<0.05, ΨΨ p<0.01 versus the corresponding group, indicated by line.

We next assessed whether *Giardia* infection may activate host AMPs. Analyses of colonic tissues revealed that MBD-3 and TFF3 mRNA was significantly higher in co-infected mice compared with those infected with *C*. *rodentium* alone (Fig 5A and 5C). AMP mRNAs were also significantly higher in mice infected with *Giardia* alone compared to uninfected controls (Fig 5A and 5C). We then visualized the protein expression of AMPs in colonic tissues using immunofluorescence. *Giardia* alone increased MBD-3 and TFF3 staining intensity in the colonic lamina propria (Fig 5B and 5D). In co-infected mice, high staining intensity of MDB-3 and TFF3 was observed on the apical surface of epithelium but also within the colonic lumen (Fig 5B and 5D). In contrast, mice infected with *C*. *rodentium* alone exhibited much lower levels of MBD-3 and TFF3, which were seen predominantly in the colonic lumen and mucosa (Fig 5B and 5D). In uninfected controls, very low levels of MBD-3 and TFF3 could be seen, mostly on the epithelial surface of colonic crypts (Fig 5B and 5D).

### *G*. *duodenalis* co-infection with EPEC increases AMPs production in cysteine protease dependent manner in human intestinal epithelial cells

**Fig 6:**
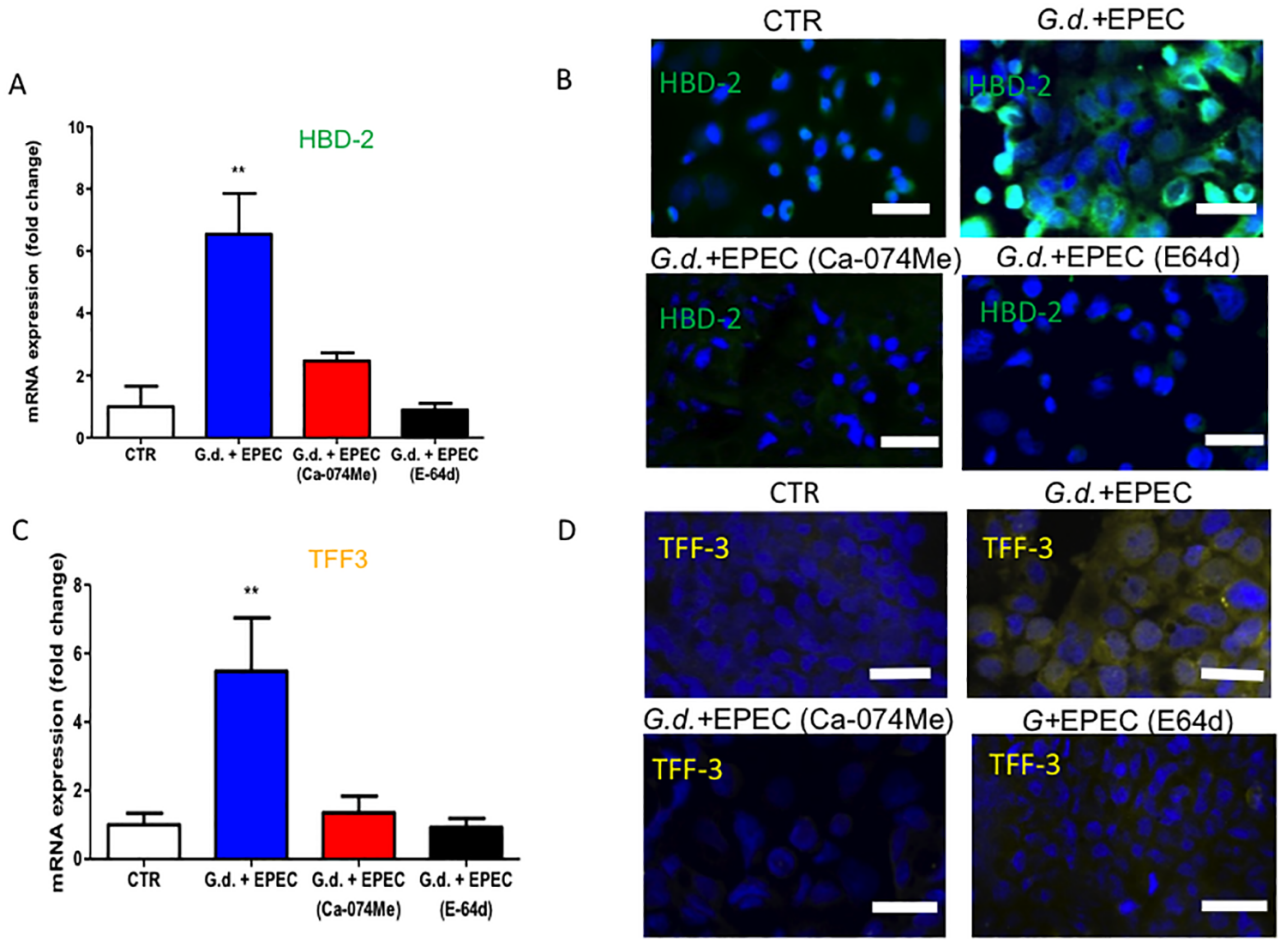
Co-infection with *G*. *duodenalis* and EPEC activates the production of anti-microbial peptides (APMs) in Caco-2 cells in cathepsin B-like protease- dependent manner. Caco-2 monolayers were co-incubated with *G*. *duodenalis* for 1 hour, pre-treated or not with a cathepsin B specific inhibitor, Ca-074Me, or a broad-spectrum cysteine protease inhibitor, E-64d, and then administered EPEC for 2 hours. (A and C) mRNA levels of HBD-2 and TFF3 were analyzed. Data is represented as fold change relative to the control group in mean ±SEM. (B and D) Immunofluorescence of Caco-2 monolayers for DAPI (blue) and HBD-2 (green) or TFF3 (yellow) was also performed. Original magnification = 400x Scale bar = 50 μm. All data are representative of n = 5–10/group and represented as mean±SEM. * p<0.05, ** p<0.01, *** p<0.001– compared to control group (CTR); *G*.*d*. means *G*. *duodenalis*.

In order to test the present *in vivo* findings from mice in human cells, confluent human Caco-2 enterocytes were co-incubated with *G*. *duodenalis* trophozoites and EPEC, the human analogue of *C*. *rodentium*. With this model we set out to determine potential mechanisms via which *Giardia* may protect a host during co-infection. In addition, we investigated whether *G*. *duodenalis* cathepsin-like cysteine proteases were involved in inducing expression of AMPs within human IECs. The expression of HBD-2 and TFF3 mRNA was significantly increased in Caco-2 monolayers co-infected with EPEC and *G*. *duodenalis* compared with those infected with EPEC alone, with *Giardia* alone, or with uninfected controls (Fig 6A and 6C, S4A and S4C Fig). Immunostaining intensity of HBD-2 and TFF3 was higher on Caco-2 enterocytes co-infected with *G*. *duodenalis* and EPEC than cells infected with EPEC alone or uninfected cells, or cells exposed to *Giardia* alone (Fig 6B and 6D, S4B and S4D Fig). Inhibition of *G*. *duodenalis* cathepsin cysteine proteases with a cathepsin B selective inhibitor (Ca-074Me) as validated recently [26], or a broad spectrum cysteine protease inhibitor (E-64d) completely abolished the elevation of mRNA and immunostained protein for both AMPs observed in co-infected cells (Fig 6B and 6D).

### *Giardia* directly inhibits the growth of A/E pathogens in a protease dependent manner

**Fig 7:**
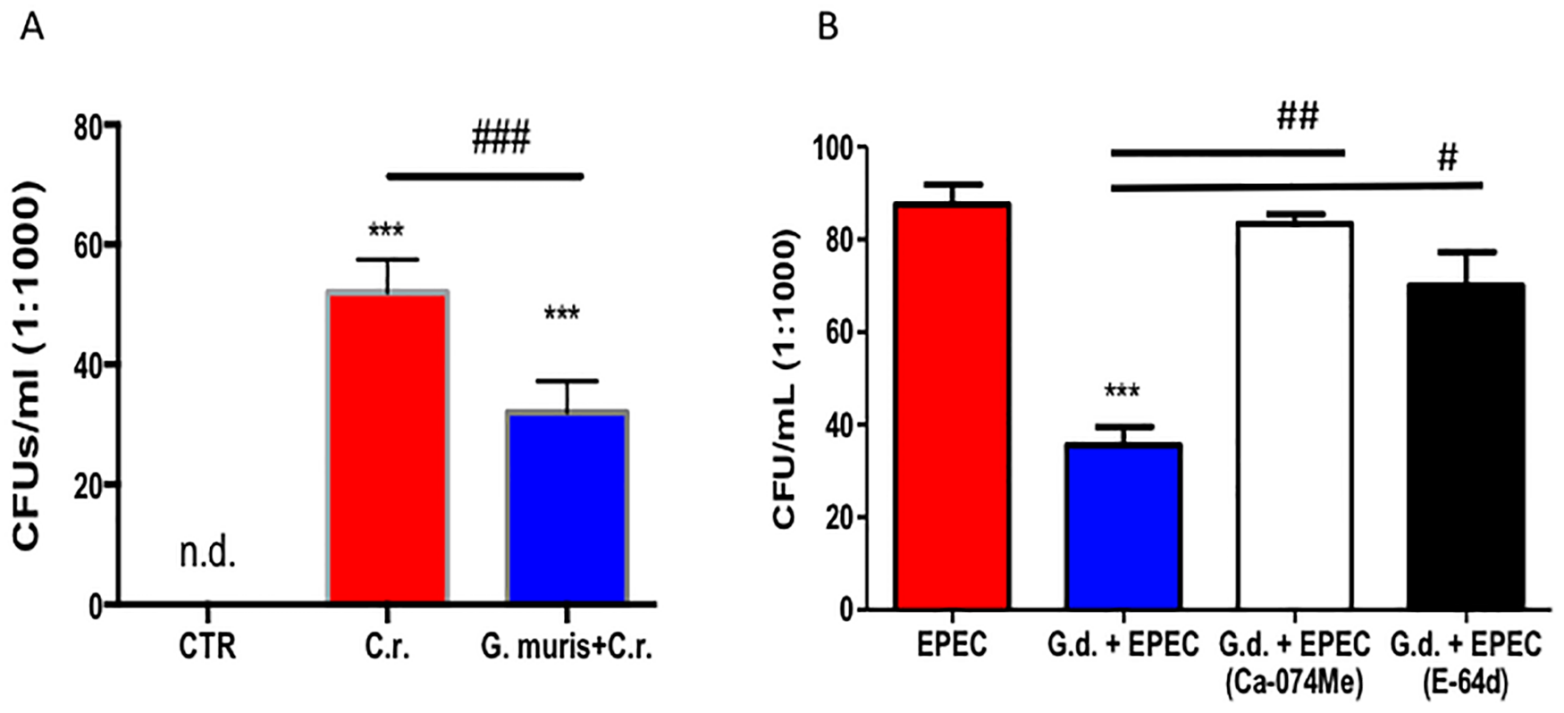
*Giardia* directly inhibits the growth of EPEC. *G*. *muris* trophozoites were co-incubated for 3h in Lysogeny broth (LB) media. Bacterial colonies were counted after plating and overnight incubation. (A) *G*. *muris* significantly reduces the growth of *C*. *rodentium*. (B) *G*. *duodenalis* significantly inhibits the growth of EPEC. All data are representative of n = 5–10/group and represented as mean±SEM. * p<0.05, ** p<0.01, *** p<0.001– compared to control group (CTR); # p<0.05, ### p<0.001 versus the corresponding group, indicated by line; n.d. means not detected, that is equivalent to 0; *G*.*d*. means *G*. *duodenalis*.

Another set of studies assessed whether *Giardia* trophozoites may directly affect A/E bacteria. Direct antibacterial properties of *G*. muris on *C*. *rodentium* and of *G*. *duodenalis* on EPEC are illustrated in Fig 7. *Giardia* significantly inhibited the growth of A/E bacteria: *C*. *rodentium* and EPEC survival *in vitro* was reduced when co-cultured for 3h with *Giardia* trophozoites (Fig 7). The inhibitory effect on EPEC by *G*. *duodenalis* was abolished when trophozoites were pre-treated with cysteine protease inhibitors (Fig 7B).

## Discussion

Our understanding of polymicrobial interactions remains incompletely understood, and the mechanisms by which such infections could either benefit or harm the host remain largely unknown. These mechanisms are commonly involved in infections due to contaminated food or water, and hence carry great significance for child health in countries with limited resources [62]. Recent studies found that in such countries, infection with *Giardia* appeared to protect children against diarrheal disease via unknown mechanisms [27, 28]. Whether *Giardia* may modulate the effects of co-infecting enteropathogens has yet to be established. The present report demonstrates that *Giardia sp*. infections may increase the expression and secretion of AMPs from IECs and protect against at least some A/E gastrointestinal pathogens during co-infection. The release of cathepsin B-like cysteine proteases from *Giardia* parasites appears to be, at least partially, responsible for inducing the expression of AMPs from IECs. The findings also show that *Giardia* cysteine proteases are able to directly inhibit the growth of an A/E bacterial pathogen. Together, these effects may explain at least in part how giardiasis may protect against intestinal disease induced by an enteropathogen.

Findings from the present study demonstrate that *Giardia* infections induce protective responses within the intestinal mucosa that attenuate the severity of disease induced by a co-infecting A/E pathogen. These findings complement previously published observations that *Giardia* infections modulate host pro-inflammatory responses to pathogenic bacteria and pro-inflammatory stimuli [26, 14, 63], and may explain how *Giardia* infections can protect against the development of diarrheal disease and reduce the expression of pro-inflammatory markers in children, when compared to children not infected with *G*. *duodenalis* [24, 25]. *Giardia* co-infections have been shown to attenuate the severity of rotavirus-induced diarrheal disease in infants [64]. In contrast, separate research has suggested that *Giardia*-rotavirus co-infections synergistically increased the incidence and severity of diarrheal disease in affected hosts [25]. Collectively, the data reveal that *Giardia* infections contain mechanisms capable of attenuating the severity of diarrheal disease induced by a co-infecting GI pathogen. These conflicting studies suggest that *G*. *duodenalis* may either enhance or attenuate the severity of diarrheal disease induced by a co-infecting GI pathogen, and highlight the need for additional research in this field.

Differences in disease outcomes may result from parasite genetics and/or genotype associated with particular *Giardia* isolates. Indeed, *G*. *duodenalis* isolates differently modulate host colonic immune responses to bacterial pro-inflammatory toxins [14] and may induce different pathophysiologic responses within hosts [65, 66]. Furthermore, the ability of *G*. *duodenalis* to alter pathophysiology may also be dependent on the type of co-infecting gastrointestinal pathogen. For example, *in vivo* experimental infections have shown that in animals co-infected with *Giardia* and *Trichinella*, presence of one parasite increases susceptibility to the other via mechanisms that have yet to be characterized [67]. *Giardia* infections have also been shown recently to shift the intestinal microbiota and change the biofilm organization of gut bacterial communities [68, 69]. Enteropathogen-induced changes of commensal microbiota are postulated to contribute to host disease [70, 71]. As shifts in the gastrointestinal microbial community have been associated with increased or decreased susceptibility to pathogenic infection [72, 73], research needs to determine whether and how *Giardia*-induced modulation of the gastrointestinal microbial community may alter susceptibility to infection with various GI pathogens.

Findings from the present study demonstrate that *Giardia spp*. infections are protective during co-infection with an A/E enteropathogen, in association with parasite-induced release of *β*-defensins and TFF3 from IECs. AMPs are essential to intestinal homeostasis and antimicrobial defense against invading GI pathogens [34]. A variety of GI pathogens, including *Salmonella spp*., *Shigella spp*., *C*. *rodentium*, EPEC and enterohemorrhagic EHEC [35–39] are known to activate AMP production. Inhibition of AMP secretion by Paneth cells enhances host susceptibility to infection [74–76]. Previous research has shown that α- and β-defensins can kill *Giardia* trophozoites *in vitro* [50]. However, the role of defensins during *Giardia* infections *in vivo* remains largely unknown. α-defensins appear to play a redundant role in controlling *Giardia* parasite burden [51], while murine β-defensin 1 is increased in animals lacking the IL-17A gene, and infected with *Giardia* parasites [77]. Our study is the first to demonstrate that *Giardia spp*. infections are capable of upregulating human β-defensin 2 (HBD-2) and mouse β-defensin 3 (MBD-3) in the presence of an A/E pathogen, and of attenuating the disease induced by a co-infecting GI pathogen. This study also shows that *Giardia* infections increase the expression of another anti-microbial peptide, trefoil factor three (TFF3). Goblet cell mucins and TFF3 play important roles in defending the intestinal mucosa against enteropathogens [60, 78, 79]. Importantly, the findings reported here also demonstrate that *Giardia* trophozoites have direct anti-bacterial effects via their cathepsin-like cysteine proteases. Mice in which intestinal antimicrobial peptides have been knocked-out are not viable [80, 81]. Additional research is warranted to assess whether the mechanisms of *Giardia spp*.-mediated resistance against A/E infections may also protect against other enteropathogens. Consistent with the present findings that showed that co-infection with *Giardia* attenuated *C*. *rodentium*-induced colonic elevation of MPO, a well-known marker of granulocyte infiltration, recent findings have reported that *Giardia* was able to cleave CXCL-8, a potent chemoattractant for neutrophils [26]. These effects may directly translate into an anti-diarrheagenic effect as it has been established that diarrhea caused by EHEC, as well as other pro-inflammatory bacterial enteropathogens, was directly induced by neutrophilic infiltration, independently of toxin production by the bacteria [82]. This may further explain why giardiasis can protect children against diarrheal disease in areas of the World with poor sanitation.

The *G*. *duodenalis* genome contains genes for numerous cathepsin cysteine proteases, and their functions are beginning to be understood [52]. Previous research has demonstrated that *G*. *duodenalis* cysteine proteases are involved in trophozoite encystation and excystation [54], cleaving intestinal epithelial structural proteins [55], and degrading pro-inflammatory chemokines [26]. Data from the present study establish that *G*. *duodenalis* co-infection with EPEC enhances intestinal epithelial beta-defensin and TFF3 production in a parasite cysteine protease-dependent manner, hence adding a novel role for *G*. *duodenalis* cathepsin-like cysteine proteases. The exact mechanisms require further clarification. Recent observations indicate that EHEC induces modest HBD-2 expression in human enterocytres *in vitro* via nuclear factor κB (NF-κB) and p38 MAPK pathways [83]. The effects of *Giardia* cathepsin cysteine proteases on these pathways to modulate AMPs during co-infection require further investigation. It has also been established that *Entamoeba histolytica* cysteine proteases activate NLRP_3_-inflammasome in macrophages [84]. Inflammasome activation is known to play a pivotal role in host defense against A/E pathogens [85], and animals deficient in NLRP3 display altered colonic β-defensin profiles [86]. The effects of *Giardia* cysteine proteases on inflammasome in the context of parasite-induced activation of AMPs is the topic of ongoing research. Microbial-microbial interactions within the gut are extremely complex, and involve the pathogen, the host, and the microbiota. As it was recently reported that *Giardia* modifies the gut microbiota [68] more research is needed to identify the role played by the intestinal microbiome in these interactions, as well as the possible difference between Giardia Assemblages A and B.

In conclusion, our study demonstrates that *Giardia spp*. attenuates the severity of intestinal disease induced by a co-infecting A/E bacterial pathogen. *Giardia* also directly inhibits the growth of pathogenic bacteria via its cysteine proteases. In addition, we propose that another mechanism leading to protection during a co-infection with A/E pathogens may involve a *Giardia* cysteine(s) protease(s)-dependent activation of host-defense via expression and secretion of AMPs such as HBD-2 and TFF3. Further research is needed to assess the molecular pathway(s) by which *Giardia* infection could activate host defensive mechanisms that leads to the activation of AMPs and TFF3 production in the presence of an A/E enteropathogen. Study of the mechanisms whereby microbial-microbial interactions may modulate disease outcome carry great potential for the development of therapeutic strategies in parts of the world with poor sanitation where A/E-induced enterocolitis is common.

## Materials and methods

### Ethics statement

All animal experiments have been approved by the Animal Care Committee at the university of Calgary (approval certificate #AC13-0067). The committee approved of the procedures described in the protocol and certifies that they are in accordance with the principles outlined in the current guidelines of the Canadian Council on Animal Care.

All isolates used in this study have been used for over 15 years in the laboratory. Therefore, their isolation did not require their approval from an ethical review board at their time of collection.

### *Giardia* parasites and bacterial cultures

*G*. *duodenalis* NF Assemblage A was obtained from a water sample during an outbreak in Newfoundland, Canada [87]. Keister’s modified TY1-S-33 media supplemented with piperacillin (Sigma-Aldrich, Oakville, Canada) was used to grow trophozoites to confluence axenically in 15 ml polystyrene tubes (Falcon, NY, USA) at 37°C [88, 89]. *G*. *muris* trophozoites were obtained from the duodenal scrapings of CD-1 mice infected with *G*. *muris* cysts (provided by Dr. Allan Shostak, University of Alberta, Edmonton, Canada). Five days after cyst inoculation, CD-1 mice were sacrificed and trophozoites were isolated from a 15 cm segment of duodenum and proximal jejunum in sterile TYI-S33 *Giardia* medium containing 3.0 mg/ml piperacillin (Sigma-Aldrich, Oakville, Canada). After vortex shaking, tube contents were filtered through sterile gauze, centrifuged at 800g for 10 minutes and pellets were incubated in fresh TYI-S33 *Giardia* medium for 2 hours. *G*. *muris* trophozoites were detached by cold PBS, pelleted by centrifugation at 800g for 10 minutes, and diluted in sterile PBS at a concentration of 5.0x10^6^ trophozoites per ml. EPEC strain O127:H6 (typical EPEC strain; provided by R. DeVinney, Microbiology and Infectious Diseases, University of Calgary) and *C*. *rodentium* (obtained from Dr. P. Sherman, Hospital of Sick Children, University of Toronto) were grown in Luria-Bertani (LB) broth (BD, Missisauga, Canada) at 37°C to log phase. Bacteria were harvested by centrifugation at 1000g for 10 minutes and re-suspended in appropriate volume of Dublecco’s PBS (Sigma-Aldrich, Oakville, Canada). GFP-labeled *C*. *rodentium* bacteria were constructed by chromosomal insertion of *gfp* (green fluorescent protein) into *C*. *rodentium* DBS-100 [61].

### Co-infection model in mice

*C*. *rodentium* co-infection with *G*. *muris* in mice was used to model EPEC co-infection with *G*. *duodenalis* in humans. Seven to eight week old C57BL/6 male mice (Charles River, Sherbrooke, Canada) were housed in facilities with 12-hour light/dark cycles and free access to food and water. Animals were infected by oral gavage with 5.0x10^6^ *G*. *muris* trophozoites in 0.1 ml of Dulbecco’s PBS (Sigma-Aldrich, Oakville, Canada). One-hour after inoculation, animals were infected with 2.5x10^8^ colony-forming units (CFUs) of GFP-*C*. *rodentium* in 0.1 ml of PBS. Mouse body weight and disease activity index (DAI) scores were recorded daily for the next 14 days. DAI included parameters of colitis/gastroenteritis (i.e. soft, liquid, solid feces condition and presence of fecal blood). After 14 days, mice were killed by cervical dislocation and *G*. *muris* trophozoites in the duodenum were enumerated using a light microscope and hemocytometer (Hausser Scientific, Horsham, USA). Animal colonic tissues were examined as below and, collected for mRNA analysis, myeloperoxidase (MPO) activity assays, bacterial colonization assays, or fixed in a 4% paraformaldehyde solution for immunofluorescence, fluorescent *in situ* hybridization, or hematoxylin and eosin staining, as described below. Whole spleen and a part of liver were also collected for bacterial translocation assays (see below).

### Macro- and microscopic colitis severity assessment

Animal colons were opened and assessed for macroscopic damage, according to a previously published score scale [90]. Criteria assessed for microscopic damage score are submucosal edema, epithelial hyperplasia, epithelial integrity, neutrophil and mononuclear cell infiltration investigated by light microscopy on hematoxylin-eosin (H&E) sections [91–93]. For each animal, macroscopic and microscopic damage score were assessed blindly by two investigators.

### Bacterial translocation assays

Total bacterial colonization of the colon was measured by plating colonic homogenates without luminal content, previously washed with sterile Dulbecco’s PBS (Sigma-Aldrich, Oakville, Canada). Proximal colonic tissue samples were weighted and collected in 2 mL Fast-Prep tubes (MP Biomedicals, Montreal, Canada) containing a mixture of 0.9–2.0 mm stainless steel beads (NextAdvance, NY, USA). Tissues samples were diluted in 1:1000 in PBS and inoculated on LB agar (BD 244520, Mississauga, Canada) plates in aerobic and anaerobic conditions, and CFUs were counted after 24 and 48 h, respectively. Spleen and liver were collected for bacterial translocation assessment as follows: tissues were homogenized using FastPrep24 (MP Biomedicals, Montreal, Canada), after were incubated similarly on LB agar plates aerobically (24 hours) and anaerobically (48 hours) at 37°C, using the AnaeroGen 2,5L packs (Thermoscientific, Ottawa, Canada). Bacterial counts were calculated by enumerating CFUs normalized to tissue or fecal weight. Plating of GFP-*C*.*rodentium* cultures from fecal homogenate were done using Columbia blood agar plates (Sigma Aldrich, Oakville, Canada) containing chloramphenicol (30μg/ml).

### RNA extraction and quantitative RT-PCR

Total RNA was extracted from proximal colon tissues samples and Caco-2 cells using a QIAGEN RNeasy Mini Kit (QIAGEN, Toronto, Canada) following the manufacturer’s instructions and retrotranscribed by RT-qPCR KIT (QIAGEN, Toronto, Canada). Real-time PCR was conducted with QuantiTect SYBR Green PCR Kit (QIAGEN, Toronto, Canada) on a Rotor Gene 3000 Cycler (QIAGEN, Corbett, Australia) and normalized to β-actin. Primers and reaction conditions for detected genes are given in S1 Table [94–97].

### Myeloperoxidase (MPO) analysis

Myeloperoxidase (MPO) activity is a well-established marker of granulocyte infiltration to assay tissue inflammation [98]. On day 14 colon was removed, distal colonic tissue samples (where the highest level of edema in this model was indicated) were weighted, and collected in 2ml Fats-Prep tubes (MP Biomedicals, Montreal, Canada) containing a mixture of 0.9–2.0 mm stainless steel beads (NextAdvance, NY, USA). Tissue samples were suspended in 50 mM potassium phosphate buffer containing 5 mg/mL hexadecyltrimethylammonium bromide (Sigma-Aldrich, Oakville, Canada) at a ratio of 50 mg tissue per 1 mL lysis buffer. Samples were homogenized using a Fast-Prep24 device (MP Biochemicals, Montreal, Canada) at speed 6.0 for 40 seconds. The resulting homogenate solution was collected into pyrogen-free 1.5mL Eppendorf tubes and centrifuged at 10,000x g for 15 minutes at 4°C. 7 μl of supernatant was added to a 96-well plate (ThermoFisher Scientific, Ottawa, Canada) with 200 μL of the reaction mixture (comprised of 0.005 g O-dianisidine (Sigma-Aldrich, Oakville, Canada), 30 mL of distilled H2O, 3.33 mL of potassium phosphate buffer, and 17 mL of 1% H2O2). Using a microplate scanner (SpectraMax M2^e^, Molecular Devices, Sunnyvale, CA, USA), three absorbance readings at 450 nm were recorded every 30 seconds. MPO activity was measured as units of activity per milligram of tissue, with 1 unit of MPO being defined as the amount required to degrade 1 μmol of H2O2 per minute at room temperature.

### Co-infection model using a human intestinal epithelial cell line

The human adenocarcinoma (Caco-2) cell line (ATCC HTB-37, Cedarlane Corporation, ON, Canada) was grown in minimum essential medium eagle media (MEME) (Sigma-Aldrich, Oakville, Canada) supplemented with 100 g/ml of streptomycin, 100 U/ml of penicillin, 200 mM L-glutamine, 5 mM sodium pyruvate, (all from Sigma Aldrich, Oakville, Canada) and 20% heat-inactivated fetal bovine serum (FBS) (VWR, Radnor, USA). Cells were used between passages 25 and 34 and kept at 37°C, 5% CO_2_, and 96% humidity for all experiments. Confluent Caco-2 monolayers growing in 6-well plates (Falcon, NY, USA) were infected with *G*. *duodenalis* NF trophozoites at a multiplicity of infections (MOI) of 10:1 for 1 hour, and exposed to EPEC at an MOI of 100:1 for additional 2 hours.

Caco-2 cells were also grown in 8-well chamber slides pretreated with poly-L-ornithine solution (Sigma-Aldrich, Oakville, Canada) to promote adhesion. Confluent Caco-2 monolayers were infected with *G*. *duodenalis* NF trophozoites at an MOI of 10:1 for 1 hour, and EPEC at an MOI of 100:1 for a further 2 hours. Following the incubation period, samples were processed for immunofluorescence (see below). In another set of experiments, *G*. *duodenalis* trophozoites were pretreated for 30 minutes with an irreversible cathepsin B specific inhibitor l-3-*trans*-(propylcarbamoyl)oxirane-2-carbonyl)-l-isoleucyl-l-proline methyl ester (Ca-074Me, Sigma-Aldrich, Oakville, Canada) or broad spectrum cysteine protease inhibitor (2*S*, 3*S*)-*trans*-epoxysuccinyl-l-leucylamido-3-methylbutane ethyl ester (E-64d, Sigma-Aldrich, Oakville, Canada) to final concentrations of 10μM or 1μM, respectively, prior to their administration and co-incubation with EPEC and confluent Caco-2 monolayers; the concentrations of inhibitors used was adapted from previously validated studies [26, 55].

### Direct antibacterial effect of *Giardia*

*G*. *muris* trophozoites isolated from mice were co-incubated in 96 wells-plate (ThermoFisher Scientific, Ottawa, Canada) with GFP-*C*. *rodentium* CFUs at an MOI of 10:1. *G*. *muris* trophozoites and GFP-*C*. *rodentium* CFUs were resuspended in 100 μL of Dublecoos’ PBS (Sigma Aldrich, Oakville, Canada). Optical density (600 nm) was recorded for 3 hours every 20 minutes on a microplate reader (BMG Labtech, Guelph, Canada). After reading 10 μL of well content was plated on LB agar plates (BD, Mississauga, Canada) in dilution 1: 1000 and incubated at 37°C, 5% CO_2_, and 96% humidity for 24 hours. The number of colonies formed were then counted and analyzed.

Plain *G*. *duodenalis* trophozoites and pretreated for 30 minutes with specific l-3-*trans*-(propylcarbamoyl)oxirane-2-carbonyl)-l-isoleucyl-l-proline methyl ester (Ca-074 Me) (Sigma-Aldrich, Oakville, Canada) and nonspecific (2*S*, 3*S*)-*trans*-epoxysuccinyl-l-leucylamido-3-methylbutane ethyl ester (E-64d) (Sigma-Aldrich, Oakville, Canada) cysteine protease inhibitors *G*. *duodenalis* trophozoites were isolated from polystyrene tubes at 100% confluency were co-incubated in 96 wells-plate with *EPEC* at an MOI of 10:1 for 2 hours. *G*. *duodenalis* trophozoites and EPEC were resuspended in 100 μL of Dublecco’s PBS (Sigma-Aldrich, Oakville, Canada). Optical density (600 nm) was recorded for 3 hours every hour on a microplate reader (BMG Labtech, Guelph, Canada). After co-incubation 10 μL well content was inoculated on LB agar plates (BD 244520, Mississauga, Canada) in dilution 1:1000 and incubated at 37°C, 5% CO_2_, and 96% humidity for 24 h. The number of colonies formed were then counted and analyzed.

### Immunofluorescence and fluorescence *in situ* hybridization (FISH)

Formalin-fixed paraffin-embedded colonic tissues were sectioned (6 μm) and analyzed via immunofluorescence and FISH. For FISH analysis, tissue sections were hybridized with 10 ng/mL of a universal bacterial 16S fluorescent rRNA probe, according to previously published protocol [99]. For immunofluorescence, tissue sections were incubated with anti-CR LPS (Biotech Laboratories, Ottawa, Canada), biotinylated goat anti-GFP (GeneTex, Irvine, USA), anti-MBD-3 (Santa Cruz Biotechnology, Dallas, USA), anti-TFF3 (Santa Cruz Biotechnology, Dallas, USA) overnight at 4°C.

Confluent Caco-2 monolayers were fixed with 100% methanol and permeabilized with 0.1% Triton 1% BSA solution in PBS. Primary antibodies were used to detect β defensin-2 (Santa Cruz Biotechnology, Dallas, USA) and TFF3 (Santa Cruz Biotechnology, Dallas, USA). The selectivity of these antibodies has been previously validated [61, 100–102]. Caco2 cells are known to produce low levels of TFF3, and the use of Caco2 enterocytes to detect modulation of TFF3 release has been previously validated [103–105]. Images were acquired using a Nikon epifluorescent microscope, and ImageJ was used for microscopic image analysis.

### Statistical analysis

Data were expressed as mean ±SEM. Data representation and statistical analysis were performed using GraphPad Prism 6 software for Macintosh (San Diego, USA). Statistical significance was determined by one-way analysis of variance with Kruskal—Wallis test or by two-way analysis. Comparison between groups was performed using Tukey’s test for multiple comparison analyses. Mann Whitney’s test was used to compare non-parametric data. An associated *P* value of less than 0.05 was considered significant.

## Supporting information

S1 Fig *Giardia* co-infection attenuates microscopic damage in the colon of mice infected with *C*. *rodentium*. Male C57/Bl6 mice (7–8 week old) were infected with *G*. *muris* and *C*. *rodentium (C*.*r*.*)* separately and in co-infection. On 14^th^ day post-infection, colonic samples were collected and stained. (A-D) **Hematoxylin eosin** (H&E) staining of colon tissues, representatives from all four groups. (Original magnification = 100x. Scale bar = 100 μm). (E-F) H&E staining of colon tissues, represents data from *C*. *rodentium*-infected mice and co-infected group. (Original magnification = 200 x. Scale bar = 50 μm).
(TIF)

S2 Fig *Giardia* prevents *C*. *rodentium* attachment, and invasion through the colonic epithelium. Male C57/Bl6 mice (7–8 week old) were infected with *G*. *muris* and *C*. *rodentium (C*.*r*.*)* separately and in co-infection. Colonic samples were collected and analyzed on 14^th^ day post-infection. Anti-GFP-*C*. *rodentium* staining represents *C*. *rodentium* burden of colon (attachment and translocation). Mucosal surface is indicated by a white dash line (Original magnification = 400x. Scale bar represents 25 μm. Red—bacteria, Green—DAPI). Arrows indicate deep crypt invasion of bacteria to host tissues. All data are representative of n = 5–10/group.
(TIF)

S3 Fig *Giardia* inhibits *C*. *rodentium* translocation to the liver. Male C57/Bl6 mice (7–8 week old) were infected with *G*. *muris* and *C*. *rodentium (C*.*r*.*)* separately and in co-infection. On 14th day post-infection, liver samples were plated on lysogeny broth (LB) agar to reveal and count translocated bacteria. All data are representative of n = 5–10/group and represented as mean±SEM. * p<0.05, ** p<0.01, *** p<0.001– compared to control group (CTR) # p<0.05 versus the corresponding group, indicated by line; n.d. means not detected, that is equivalent to 0.
(TIF)

S4 Fig *Giardia* co-infection with EPEC activates AMPs production in Caco-2 cells in a cathepsin B-like -dependent manner. *G*. *duodenalis* co-infection with EPEC increased mRNA expression of HBD-2. Cells were incubated with *G*. *duodenalis* for 3 hours and co-incubated with EPEC for 2 hours. (A) mRNA level of HBD-2. (B) IF staining for HBD-2. (C) mRNA level of TFF3. (D) IF staining for TFF3. Data is represented as fold change means relative to the control group ± SEM. (Original magnification = 400x. Scale bar = 25 μm. Blue-DAPI, Yellow-HBD-2, Green-Human TFF3). All data are representative of n = 5–10/group and represented as mean±SEM. * p<0.05, ** p<0.01, *** p<0.001– compared to control group (CTR); *G*.*d*. means *G*. *duodenalis*.
(TIF)

S1 Table Primers sequences for PCR. (DOCX)
