# Supplementary figures and images for: Giardia co-infection promotes the secretion of antimicrobial peptides beta-defensin 2 and trefoil factor 3 and attenuates attaching and effacing bacteria-induced intestinal disease
Source: PLoS One. 2017 Jun 16;12(6):e0178647. doi: 10.1371/journal.pone.0178647 (PMC5473565; doi:10.1371/journal.pone.0178647)

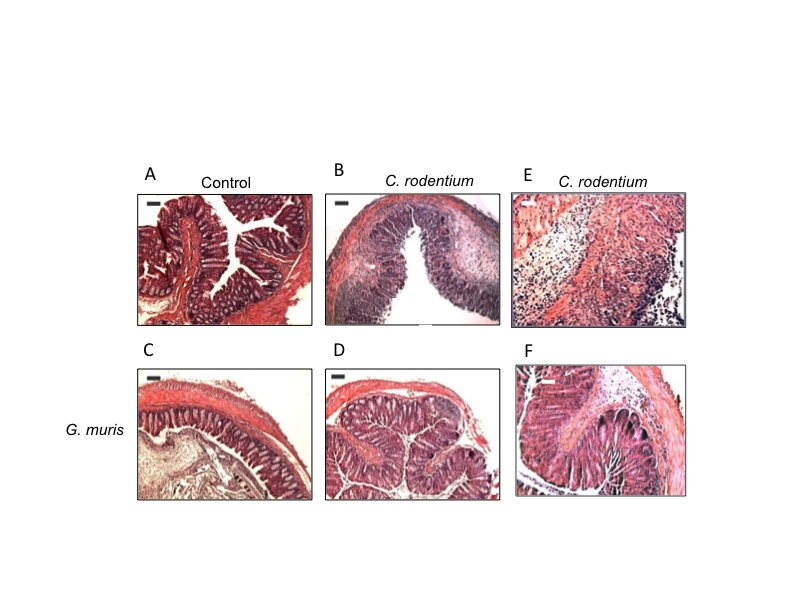

Supplement: S1 Fig — Male C57/Bl6 mice (7–8 week old) were infected with G. muris and C. rodentium (C.r.) separately and in co-infection. On 14th day post-infection, colonic samples were collected and stained. (A-D) Hematoxylin eosin (H&E) staining of colon tissues, representatives from all four groups. (Original magnification = 100x. Scale bar = 100 μm). (E-F) H&E staining of colon tissues, represents data from C. rodentium-infected mice and co-infected group. (Original magnification = 200 x. Scale bar = 50 μm). (TIF) [file pone.0178647.s001.tif]

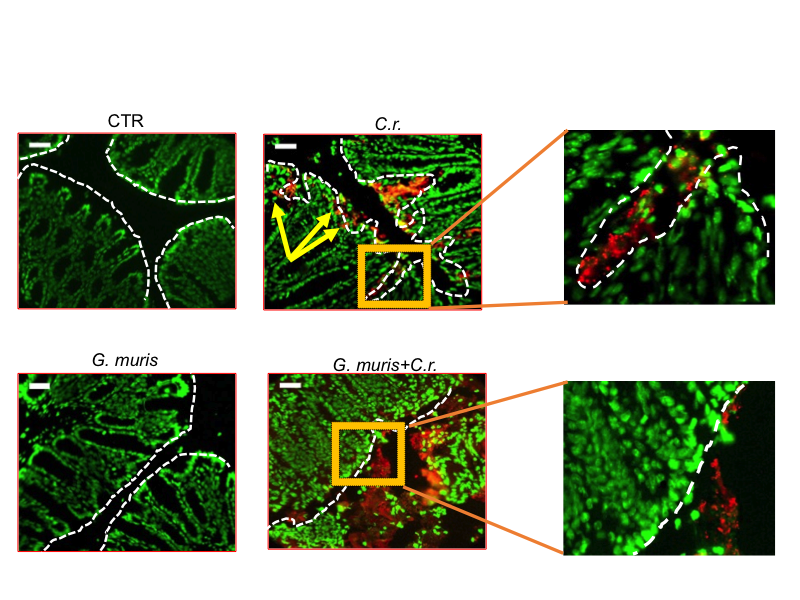

Supplement: S2 Fig — Male C57/Bl6 mice (7–8 week old) were infected with G. muris and C. rodentium (C.r.) separately and in co-infection. Colonic samples were collected and analyzed on 14th day post-infection. Anti-GFP-C. rodentium staining represents C. rodentium burden of colon (attachment and translocation). Mucosal surface is indicated by a white dash line (Original magnification = 400x. Scale bar represents 25 μm. Red—bacteria, Green—DAPI). Arrows indicate deep crypt invasion of bacteria to host tissues. All data are representative of n = 5–10/group. (TIF) [file pone.0178647.s002.tif]

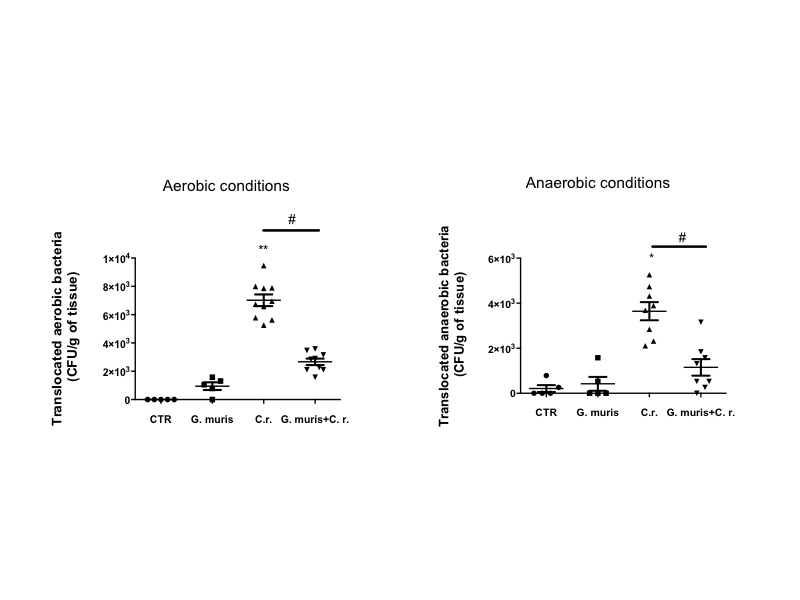

Supplement: S3 Fig — Male C57/Bl6 mice (7–8 week old) were infected with G. muris and C. rodentium (C.r.) separately and in co-infection. On 14th day post-infection, liver samples were plated on lysogeny broth (LB) agar to reveal and count translocated bacteria. All data are representative of n = 5–10/group and represented as mean±SEM. * p<0.05, ** p<0.01, *** p<0.001– compared to control group (CTR) # p<0.05 versus the corresponding group, indicated by line; n.d. means not detected, that is equivalent to 0. (TIF) [file pone.0178647.s003.tif]

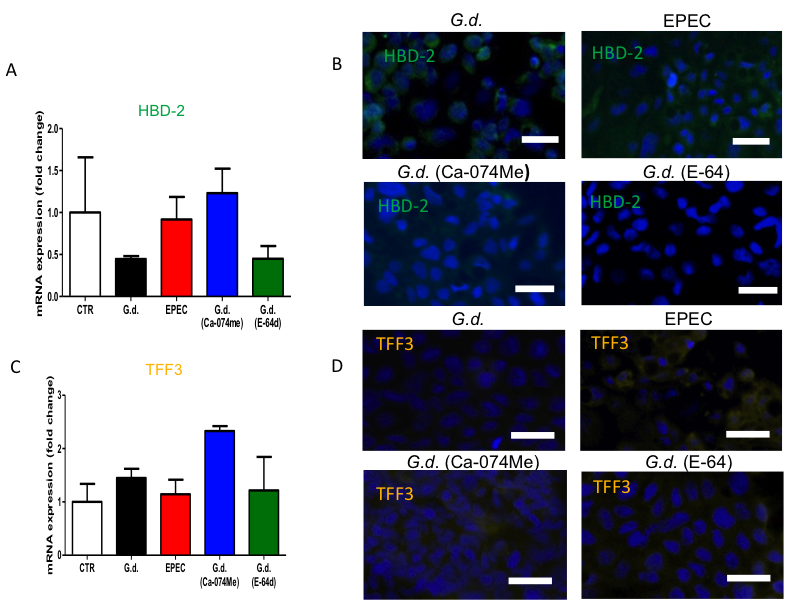

Supplement: S4 Fig — G. duodenalis co-infection with EPEC increased mRNA expression of HBD-2. Cells were incubated with G. duodenalis for 3 hours and co-incubated with EPEC for 2 hours. (A) mRNA level of HBD-2. (B) IF staining for HBD-2. (C) mRNA level of TFF3. (D) IF staining for TFF3. Data is represented as fold change means relative to the control group ± SEM. (Original magnification = 400x. Scale bar = 25 μm. Blue-DAPI, Yellow-HBD-2, Green-Human TFF3). All data are representative of n = 5–10/group and represented as mean±SEM. * p<0.05, ** p<0.01, *** p<0.001– compared to control group (CTR); G.d. means G. duodenalis. (TIF) [file pone.0178647.s004.tif]
