# Supplementary material for: Giardia co-infection promotes the secretion of antimicrobial peptides beta-defensin 2 and trefoil factor 3 and attenuates attaching and effacing bacteria-induced intestinal disease
Source: PLoS One. 2017 Jun 16;12(6):e0178647. doi: 10.1371/journal.pone.0178647 (PMC5473565; doi:10.1371/journal.pone.0178647)
Supplement: S1 Table — (DOCX) [file pone.0178647.s005.docx]

Gene of Forward gene Reverse gene

interest

Murine β-defensin 3 5-GCATTGGCAACACTCGTCAGA-3 5-CGGGATCTTGGTCTTCTCTA-3

(MBD-3)

Murine Trefoil factor three 5′-CAGATTACGTTGGCCTGTCTCC-3′ 5′-ATGCTTGCTACCCTTGGACCAC-3′

(TFF-3)

Human β-defensin 2 5-AGCCTAGCAGCTATGAGGATC-3 5-CTTCGGCAGCATTTTGCGCCA-3

(HBD-2)

Human Trefoil factor three 5-GTGCCAGCCAAGGACAG-3 5- CGTTAAGACATCAGGCTCCAG-3

(TFF-3)

Murine β-actin 5-TGTGATGGTGGGAATGGGTCAGAA-3 5-TGTGGTGCCAGATCTTCTCCATGT -3

|  |  |  |
| --- | --- | --- |

Human β-actin 5′-GTGGGCCGCTCTAGGCACCAA-3′ 5′-CTCTTTGATGTCACGCACGATTTC-3′

- In all PCR experiments there was an initial denaturation step of 95°C for 5 min before PCR cycling. For end point PCR experiments there was an extension step of 72°C for 10 min after the final cycle.

- - MBD-3 primer sequences were obtained from reference 63.

- Murine Tff3 primer sequences were obtained from reference 64.

- - HBD-2 primer sequences were obtained from reference 65.

- Human Tff3 primer sequences were obtained from reference 66.

- - Murine β-actin primer sequences were obtained from reference 64.
- - Human β-actin primer sequences were obtained from reference 66.
